# Nutrition knowledge and practice of midwives in Botswana

**DOI:** 10.4102/hsag.v29i0.2589

**Published:** 2024-08-30

**Authors:** Anastacia Masesane, Thembekile Dhlamini, Maria Nnyepi, Xikombiso Mbhenyane

**Affiliations:** 1 Division of Human Nutrition, Faculty of Medicine and Health Sciences, Stellenbosch University, Cape Town, South Africa; 2 Department of Health Services Management Ministry of Health, Gaborone, Botswana; 3 Faculty of Education, University of Botswana, Gaborone, Botswana

**Keywords:** knowledge, practices, pregnancy, maternal nutrition, midwives

## Abstract

**Background:**

The roles and responsibilities of midwives include providing adequate nutrition assessment, nutrition and health education, counselling, and support to pregnant women.

**Aim:**

This study aims to assess midwives’ nutrition knowledge and to what extent they integrate maternal nutrition in services provided at health facilities.

**Setting:**

This study included hospitals and clinics within the three selected districts in Botswana.

**Methods:**

A cross-sectional study design with an analytical component was employed. Direct observation through a checklist and a structured interviewer-administered questionnaire were used. Data were analysed using SPSS IBM version 26.

**Results:**

A sample of 102 midwives participated, resulting in a response rate of 82%. Most of the participants were females (89.2%). Maternal nutrition knowledge was found to be variable but decreased with midwives’ age. A statistically significant correlation coefficient of *p* < 0.005 at *r* = –0.278 was observed between maternal nutrition knowledge and age. Similarly, there was a statistically significant negative correlation between maternal nutrition knowledge, practices and maternal nutrition course attended using Pearson correlation (*r* = –0.217 *p* < 0.028).

**Conclusion:**

Midwives had *adequate to variable* but declining maternal nutrition knowledge and practices with age. There is a need to provide midwives with refresher courses, as their nutrition knowledge and practices were related with courses attended.

**Contribution:**

The study contributes to provide the literature concerning nutrition knowledge and practices of midwives. The results will assist in addressing the gaps encountered and lead to the improvement of maternal nutrition and pregnancy outcomes.

## Introduction

Inadequate nutrition during pregnancy is reported to have adverse effects on both the mother and the foetus, particularly at the early developmental stages of the foetus (World Health Organization [WHO] 2014). The adverse effects of poor maternal nutrition, if not addressed, may ultimately lead to maternal and child mortality (WHO 2014). Some examples of the adverse effects of poor maternal nutrition include intra-uterine retardation, poor brain development, increased congenital disabilities, infection risk, premature birth, and infant mortality (Nnam 2015). According to the 2019 United Nations (UN) report on levels and trends in child mortality, 45% of the child deaths are nutrition related (UNICEF et al. 2019). On the contrary, the Global Scorecard Report 2021 revealed that inadequate breastfeeding contributes to 16% of child deaths each year. Midwives and nurses play a vital role in facilitating mothers to protect, promote, and support breastfeeding for the first 2 years to save lives (Global Breastfeeding Score Card 2021). Optimal nutrition during pregnancy is vital for the production and supply of breastmilk. However, midwives’ commitment to supporting breastfeeding, among other provisions of maternal nutrition, is yet to be established. It is important to emphasise that child mortality because of nutrition deficiencies can be prevented by tackling the matter from the foundation stage and providing relevant nutrition education to expecting women.

Adequate nutrition throughout the stages of pregnancy is the key to a positive pregnancy outcome. Several studies indicate that a balance of the macro- and micro-nutrients during pregnancy is necessary to promote appropriate gestational weight gain and proper foetal growth and development (Parisi et al. 2019). To prevent the risk of anaemia during pregnancy, the WHO recommends daily supplementation of iron and folic acid, containing 30 or 60 mg of elemental iron and 0.4 mg of folic acid (WHO 2020). A study in India on maternal vitamin B12 status during pregnancy revealed that higher risk of neural tube defects (NTD), low birth weight, and gestational diabetes during pregnancy were associated with vitamin B12 deficiencies (Behere et al. 2021):

- To mitigate the risks of nutrient deficiencies outlined earlier in the text, it is evident that adequate and relevant nutrition education during pregnancy is critical. Healthy diets that include the most diverse and nutrient-rich foods provide maternal and foetal health and reduce the prevalence of low birthweight. Midwives are best positioned to provide nutrition education and counselling to pregnant women through antenatal care (ANC). Therefore, their nutrition knowledge and the quality of nutrition education provided as front-line health workers to pregnant women are a priority. Arrish et al. indicated that even though the importance of nutrition during pregnancy is acknowledged by midwives, pregnant women often receive inadequate nutrition education (Arrish, Yeatman & Williamson 2016). Similarly, a review on nutritional education for midwives found that 55% of the respondents indicated that nutritional instruction in midwifery training was insufficient and lacked a practical approach (Olloqui-Mundet et al. 2023). In Botswana, few studies have been undertaken to ascertain midwives’ nutrition knowledge and practices and the general effects of these attributes on nutrition education for pregnant women. Hence, this study builds on this work by assessing midwives’ nutrition knowledge and practices and the integration of maternal nutrition care into maternal health services in Botswana. This study aimed to assess midwives’ nutrition knowledge and practices and to determine to what extent they integrate maternal nutrition in maternal health services at the health facilities in the selected districts in Botswana. To achieve this aim, the specific objectives included establishing the knowledge of midwives on maternal nutrition services, investigating their practices towards providing maternal nutrition services, and exploring the relationship between nutrition knowledge, practices, and socio-demographic characteristics of midwives.

## Research methods and design

### Study design and setting

A cross-sectional descriptive quantitative study design was employed. The selected methodology is intended to achieve the set objectives of the study and answer the research question: ‘*Are midwives knowledgeable about providing appropriate maternal nutrition during maternal health services at health facilities?*’ (Kesmodel, 2018). The South East, Kanye, and Kgatleng health districts were conveniently selected. All the health facilities providing maternal health services at the selected health districts were included. The health facilities comprised three primary hospitals, eight clinics with maternity services, and 26 clinics with general sexual and reproductive health services.

### Study population and sampling strategy

The District Health Management Team (DHMT) provided the number of midwives employed and the list of all health facilities within the three health districts. There were approximately 181 midwives in total. Out of 181, the sample needed for the study was 124 midwives. A 95% confidence level and *p* < 0.05 statistical significance difference were used as a basis for sample calculation. Based on the number of midwives per district, all-inclusive sampling was employed, targeting all midwives who met the criteria. The method was used because the study targeted specific attributes of midwives as they provide antenatal care services. The participants were recruited through official communication from the DHMT, which informed health facilities about the study and urged those available during the study period to voluntarily participate. Midwives were briefed by the researcher about the study and procedures during morning briefs, and all those interested in taking part submitted their names to the researcher to determine eligibility and enrolment. All midwives meeting the inclusion criteria, available on the day of data collection and willing to participate were enrolled in the study. In total, 102 midwives volunteered to participate in the study. The response rate was 82%.

### Data collection

A quantitative data-collection method was employed, using direct observation through a checklist and a structured interviewer-administered questionnaire (Online Appendix 1). The development of a structured questionnaire and observation checklist was aligned with the ‘WHO recommendations on antenatal care for a positive pregnancy experience’ (WHO 2020). The questionnaire was adapted from the previously validated questions for similar studies (Arrish et al. 2016). The Institute of Health Sciences Gaborone verified the questionnaire to ensure it aligned with the local midwifery curriculum. The questions that did not apply to the local setting were also removed and replaced with others that were more culturally appropriate. Mornings were used for observation because the ANC took place during that time. The primary investigator observed midwives without asking any questions, and every correct practice that the midwives performed during antenatal care received a tick. The observed sections focused on the maternal nutrition practices and nutritional assessments conducted during ANC. This took approximately 30 min of the consultation period.

The interviewer conducted the structured interviews in the afternoons while midwives had fewer clients to attend to, and the interviews lasted about 30 min. It was administered in English, one of the official languages, and is easily understood by health professionals. The entire data-collection process for the study took approximately 2 months between March and June 2020.

### Reliability and validity

The study ensured that the instrument complied with both content and face validity. Two midwives from Institute of Health Sciences Gaborone validated the questionnaire, while another set of professionals, including two midwives, a dietitian, and a nutritionist from the Ministry of Health, also participated in the validation process. Questions not applicable to the local context were removed. The piloting of the study covered 5% of the study sample within the same study setting to assess the reliability. This included data collection involving practising midwives, data entry, and analysis of the data. A statistician was involved in guiding methods and statistical analysis throughout the process.

### Constructs measured

The study measured the socio-demographic characteristics of midwives, nutrition knowledge, and maternal nutrition practice. The direct observation section comprised 52 questions. The responses were scored based on the maternal nutrition practice performed using the checklist. The socio-demographic section consisted of 20 questions that were administered through an administered questionnaire. The knowledge questions also consisted of 20 questions, of which 13 had one correct answer, and therefore, only one answer had to be selected. Seven were multiple-choice questions with more than one correct answer. The single-answered items were scored based on midwives’ knowledge of the answer, allocated a score of one or zero if they did not know (Arrish et al. 2016). The seven questions with more than one correct answer had three correct answers, and each answer was allocated a mark, totalling 21 marks for the seven questions (Arrish et al. 2016). The practice section consisted of 12 questions and was scored according to the response. Answers indicating responses of ‘yes’ and ‘sometimes’ were considered positive and scored as ‘1’ while a response of ‘no’ was considered negative and scored as ‘0’ (FAO 2014). The practice of midwives was therefore assessed in two forms through an observation checklist and the questionnaire.

The scoring categorisation of the questionnaire was derived from a similar study on pregnancy nutrition knowledge and experiences of pregnant women and antenatal care clinicians in Australia (Lee et al. 2018). In this study, adequate knowledge or well-known was shown by a score of 80% and above while moderate knowledge was classified by 50% – 79%. Poor knowledge was classified by a score of below 50% among clinicians.

### Data analysis

Data were collected, captured, and analysed using the statistical package for social science (SPSS) IBM version 26. Descriptive statistics were used to analyse demographic characteristics such as age, sex, education level, professional qualification, years of experience, nutrition knowledge, and practices. Direct observation was analysed using simple univariate analysis. The normality of data was tested through frequency distribution. Tables and graphs were used to present data as frequencies, means (standard deviations), and percentages. The association between knowledge and socio-demographic characteristics was determined through Pearson’s correlation. The statistical difference in knowledge and practices between the two groups (gender) was determined through an ANOVA test. Statistically significant differences were identified at *p* < 0.05 and a confidence level of 95%.

### Ethical considerations

Ethical approval was obtained from the Stellenbosch University Health Research Ethics Committee of the Faculty of Health Sciences (Ref: S19/10/224). Further approval was attained from the Health Research and Development Committee of the Botswana Ministry of Health (Ref: HPDME 13/18/11). The permission to access the health facilities was obtained from the District Health Management Teams (Ref: DHMT/K/112 I).

The study was classified as low risk. Prior to data collection, verbal and written consent was obtained from all midwives. The objectives of the study were explained, and their questions were answered. A written consent form was signed by midwives before participating in the study. Participation was voluntary, and without prejudice, they were further informed that they were free to withdraw from the study at any time. They were also assured of confidentiality and were assigned codes, and no names were used. Information on the data collected and data analysis remained in the researcher’s password-protected computer and stored in accordance with the guidelines of the Research Ethics Committees.

## Results

### Sample characteristics

The sections assessed included socio-demographic characteristics of midwives, nutrition training background, nutrition knowledge, and their practices as they provided ANC services. The participants’ mean age was 48.2 years old. Most of the midwives were females, with only 10.8% as males with midwifery years of experience ranging from 2 to 38 years. Table 1 presents the socio-demographic characteristics of the midwives.

**TABLE 1:** Socio-demographic characteristics of midwives (*N* = 102).

Characteristics	Frequency	%
**Gender**		
Male	11	10.8
Female	91	89.2
**Level of education**		
Diploma in Nursing	91	89.2
Degree in nursing, community, psychiatry and public health	11	10.8
**Midwifery qualification**		
Diploma in midwifery	92	90.2
Advanced diploma in midwifery	8	7.8
Certificate in midwifery	2	2.0
**Current position**		
Principal Registered Nurse-midwife	89	87.2
Chief Registered Nurse-midwife	5	4.9
Senior Nursing Officer	4	3.9
Nursing Officer I	2	2.0
Registered Nurse-midwife	1	1.0
Principal Nursing Officer II	1	1.0
**Years of experience**		
Less than 5	10	9.8
6–10	24	23.5
More than 10	68	66.7

### Nutrition training background of midwives

Midwives received information about basic nutrition (64.7%) and maternal nutrition (74.5%) during their midwifery training. Insufficient maternal nutrition education was reported by midwives with no basic nutrition training.

Midwives indicated inadequate opportunities to enrol for continuing education for nutrition-related courses as part of on-the-job training, while 10% reported having attended maternal nutrition courses in the past 2 years. Some of the training attended by 5.9% of midwives included maternal and child nutrition, while Prevention of Mother to Child Transmission (PMTCT), Infant and Young Child Feeding (IYCF) training was attended by 3.9%, and growth monitoring and promotion (GMP) by only one of midwives. The details of the Nutrition Training Background of Midwives are shown in Table 2.

**TABLE 2:** Basic nutrition and maternal nutrition module (*N* = 102).

Content	Frequency	%
**Basic nutrition training**		
Yes	66	64.7
Maternal nutrition training	76	74.5
Maternal nutrition courses provided on the job	11	10.8
**Nutrition related courses attended**		
Maternal and child nutrition	6	5.9
PMTCT and IYCF	4	3.9
GMP	1	1.0

PMTCT, prevention of mother-to-child transmission; IYCF, infant, young and child feeding; GMP, growth monitoring and promotion.

### Maternal nutrition knowledge of midwives

**FIGURE 1:**
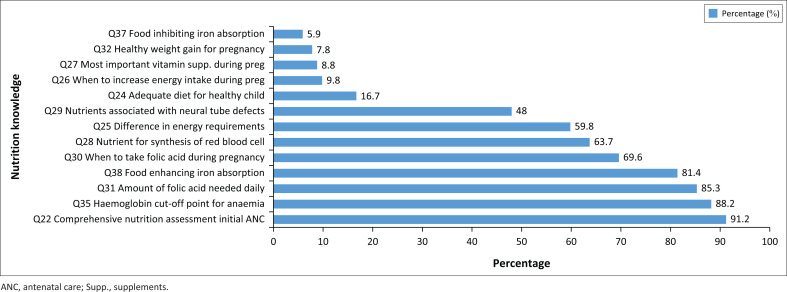
Correct responses of single answered questions.

Figure 1 depicts a summary of responses for single-answered questions on nutrition knowledge. The findings revealed that most midwives (91.2%) regarded the initial ANC visit as the most critical for conducting an extensive nutritional assessment. Over half of the midwives (52%) indicated that pregnant mothers should have an adequate diet throughout pregnancy. Only a few midwives (16.7%) understood the importance of adequate diet during pregnancy, before conception and throughout pregnancy until lactation duration to ensure a healthy infant.

Almost 59.8% of midwives knew there was a difference in the energy requirement during the three trimesters. On pregnant mothers’ energy requirements, some midwives reported an increase in energy requirements during pregnancy, while some midwives reported an energy increase in the first trimester; a few midwives (9.8%) responded that there are increased energy requirements during the second and third trimesters in pregnancy.

Figure 1 depicts midwives’ nutrition knowledge using different single questions. Midwives answered by giving the correct words. Just above half of midwives (55.9%) were not familiar with essential supplements for vegetarian pregnant women. Vitamin B12 was specified by a few midwives (8.8%) as an essential supplement for vegetarian pregnant women. Almost half of the midwives (48%) understood that folic acid was strongly associated with preventing neural tube defects. Most midwives (69.6%) demonstrated a good knowledge of the appropriate period for taking folic acid, indicating that pregnant women should take folic acid supplements before conception and throughout the first trimester. The overall average knowledge for midwives given the correct responses was 49%.

### Nutrition knowledge and relationship with socio-demographic characteristics

A statistically significant negative correlation (*p* = 0.005) was shown by a Pearson correlation coefficient between age and midwives’ maternal nutrition knowledge. As the age of midwives increased, their knowledge decreased, indicating a relatively weak negative correlation of –0.278. No significant association (*p* = 0.455) was shown between midwives’ level of education and maternal nutrition knowledge (Table 3). However, the highest attained education level and nutrition knowledge showed a positive but weak correlation of 0.075. Years of midwifery experience and maternal nutrition knowledge of midwives also showed a significant negative correlation (*r* = –0.217, *p* = 0.028).

**TABLE 3:** Association between maternal nutrition knowledge and socio-demographic characteristics (*N* = 102).

Variables	Pearson correlation	Significance (*p*-value)	Interpretation
Maternal nutrition knowledge versus age of midwives	−0.278	0.005	**Significantly negative correlation**
Maternal nutrition knowledge versus highest education	0.075	0.455	No significant correlation
Maternal nutrition knowledge versus midwifery qualification	−0.161	0.106	No significant correlation
Maternal nutrition knowledge versus years of midwifery experience	−0.217	0.028	**Significantly negative correlation**

Note: Bold signifies significance or importance.

### Midwives’ maternal nutrition practices

Midwives were assessed through a questionnaire and direct observation of their maternal nutrition practices. Nutrition education was intentionally provided to pregnant women as they came for ANC visits. More than half (55.9%) of the midwives revealed that the importance of healthy eating was always taught to pregnant women. An increase in fruit and vegetable consumption was emphasised by 42.2% of the midwives, while a few (16.7%) indicated consumption of wholegrain foods or fibre-rich diets. Education on the reduction of unhealthy foods such as those high in saturated fats, sugar, and high salt was emphasised by very few midwives (< 3%). Some of the noted advice midwives shared with pregnant women included increasing calcium-rich foods and at least drinking eight glasses of water per day. The results showed that 50.0% of the midwives provided information on healthy eating during nutrition education and counselling sessions. However, the importance of educating pregnant women about the inclusion of iron-rich food in their diet during pregnancy was highlighted by more (69.6%) midwives. This shows a good practice for preventing anaemia during pregnancy. Giving information on the advantages and disadvantages of exclusive breastfeeding and formula feeding was reported by 84.3% of midwives. The decision on the method of feeding was left to the pregnant woman.

Information regarding nutrition supplements revealed that some midwives (52.0%) did not issue calcium gluconate to pregnant women, whereas few (20.6%) reported always giving calcium gluconate to pregnant women. Encouraging pregnant women to take nutritional supplements, including iron and folic acid, was reported by 96.1% of midwives. On the contrary, direct observation revealed that less than half (44.2%) encouraged pregnant women to take their supplements. The overall average response score for the nutrition practices of midwives was 60%.

### Direct observation versus interview on nutrition practices

**FIGURE 2:**
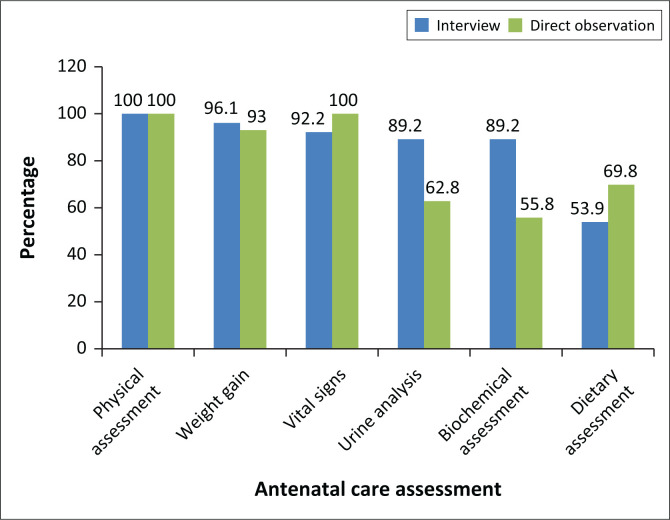
Nutrition practices direct observation versus administered interview.

Midwives fervently conducted antenatal assessments as pregnant women visited the health facility for their maternal health services while the primary investigator observed. It was observed that physical evaluation from head to toe was successfully provided by all midwives (100%). However, some of the variables showed a slight difference between direct observations and self-reported interviews. Even though only 93% of the midwives were observed assessing weight during ANC, 96.1% reported that they always assessed weight during the administered interview. Only 62.8% were observed conducting urine analysis, while 89.2% reported always conducting it during the interview, as indicated in Figure 2. Urine analysis is among the vital assessments for early detection of diabetes, among other disorders during pregnancy.

## Discussion

The study’s findings revealed that midwives lacked nutrition knowledge as the majority (52%) had poor knowledge of maternal nutrition while their maternal nutrition practice appeared to be moderately provided. The results showed that strengthening maternal nutrition among midwives needs to be prioritised. This is more critical, particularly for a country like Botswana, battling with high maternal mortality, high prevalence of low birth weight (LBW), low exclusive breastfeeding rates, anaemia during pregnancy, and non-communicable diseases.

Almost half of the participants revealed that their training did not adequately provide them with nutrition knowledge to effectively conduct maternal nutrition education to pregnant women during ANC visits. Similar findings were reported by Arrish, Yeatman and Williamson (2017) whose study on nutrition education by Australian midwives found that limited nutrition education was covered during midwifery training, mostly the basics (Arrish et al. 2017). Given the study results, the researchers recommended that the midwifery curriculum of nursing training institutions in Botswana be reviewed to close the identified gaps and address issues that might be contributing to inadequate knowledge.

One interesting finding from the study was the association of midwives’ age and nutrition knowledge. The study results showed a significant negative correlation (*p* = 0.005) between age and nutrition knowledge of midwives. It was shown that younger midwives were more knowledgeable than the older ones, which seems to be a novel contribution to nutrition knowledge of midwives’ literature. The likelihood of younger midwives being more knowledgeable could be attributed to the fact that: (1) they completed training more recently compared to the older ones, and hence, they could remember better; (2) they could have benefitted more from the reviewed training curriculum over the years with updated content; and (3) the older midwives situation was exacerbated by a lack of refresher courses, which was specified to be a disadvantage given their completion period. Therefore, it is necessary to capacitate midwives to provide appropriate maternal nutrition as less than 10% had the opportunity to attend post-training nutrition-related courses. Considering that 66.7% of the midwives had at least 10 years of experience, which approximately translates to the years they last undertook midwifery training, in-service training on maternal nutrition is highly recommended. Similar findings were reported by the study on healthy eating in pregnancy aimed at educating midwives, which found that more than 70% of midwives had over 10 years of experience (Othman et al. 2020).

The study also revealed a lack of knowledge in several areas related to maternal health. One such area was healthy weight gain during pregnancy. Only a few (7.8%) midwives correctly gave the expected healthy weight gain range as 11.5–16 kg, while the majority indicated 10–12.5 kg. Appropriate weight gain during pregnancy has been reported to contribute significantly to the infant’s birth weight (Tela, Bezabih & Adhanu 2019). Tela et al. (2019) in their study determining the effects of pregnancy weight on infant birth weight in Northern Ethiopia found that poor weight gain was related to negative intra-uterine foetal growth and, ultimately, LBW, putting infants at risk of poor breastfeeding, inappropriate child development, and poor child survival (Tela et al. 2019). Lack of understanding of the correct weight gain by most midwives in the study places pregnant women at risk of improper guidance to improve their nutritional status. The UNICEF-WHO 2019 report on LBW estimates indicated that Botswana has a high prevalence of LBW (15.6%). Attention to address this gap by having regular in-service training for midwives is recommended (WHO & UNICEF 2019). It is important to note that these results were not peculiar to Botswana. A systematic review by Olander et al., evaluating health care professional training regarding gestational weight gain, discovered that midwives and other health professionals lacked the knowledge to support gestational weight gain (Olander, Hill & Skouteris 2021). The results therefore show that regular training sessions to improve health professionals’ confidence and knowledge of gestational weight gain are critical.

Another critical area that midwives rarely attend to during ANC visits for optimal child growth and weight gain is the energy requirements. Even though midwives acknowledged a variance in the energy requirements throughout the different trimesters, only a few (10%) correctly responded that an energy increase is recommended from the second to third trimesters. Studies reported that the required energy and balanced physical activities support pregnancy needs and the appropriate development of the foetus (Most et al. 2019). Therefore, applicable guidance on energy requirements during ANC plays a vital role.

The study revealed inadequate knowledge of folic acid requirements during pregnancy. Only 48% of midwives were knowledgeable that the nutrient associated with preventing neural tube defects was folic acid. However, midwives knew when a pregnant woman should take this nutrient. There was a discrepancy between the daily amount of folic acid recommended for pregnant women by the midwives who participated in the study and the WHO-recommended amount. While the WHO recommends 400 mcg daily, midwives indicated 5000 mcg as the daily amount of folic acid for pregnant women. The study considered 400 mcg and 5000 mcg as acceptable folic acid daily doses. This was consistent with a study by Mbhenyane and Cherane, which investigated the consumption of iron and folate supplements among pregnant women in Mafikeng, Northwest Province, South Africa. Their study reported that the South African Department of Health protocol recommends a daily folic acid dosage of 5000 mcg (Mbhenyane & Cherane 2017).

The study findings demonstrated that midwives were knowledgeable about iron requirements for pregnant women and available food sources rich in iron. Publicity posters illustrating the importance of iron consumption during pregnancy were available in consultation rooms at the health facilities. These posters contributed to midwives’ knowledge of iron requirements and different iron-rich food sources consistently shared with pregnant women. Another nutrient that midwives demonstrated high knowledge of was the purpose of giving vitamin C. Midwives were aware that vitamin C enhanced iron absorption in the body. However, less knowledge was demonstrated on iron absorption inhibiting food items. Only a few (6.0%) of the midwives correctly listed caffeine-containing food items as food that must be avoided simultaneously with iron supplements or immediately after consuming a meal rich in iron. A study by Kumera et al. on the prevention of anaemia during pregnancy strongly recommended that midwives provide advice to pregnant women to avoid taking caffeine food sources such as coffee during pregnancy or to avoid taking such food sources together with their meals (Kumera et al. 2018).

Lastly, this study observed that the midwives performed moderately well concerning maternal nutrition practices. However, it was observed that some activities were not consistently performed. Urine analysis is critical for the early detection of diabetes, for instance, but it was not done for every pregnant woman who came for ANC. The reason for such inconsistency was the shortage of dipsticks within the health facilities. Therefore, priority was given to those pregnant women at risk of high blood pressure. Dietary assessment of pregnant women was performed moderately. Dietary assessments during ANC were undertaken by just over half of the midwives. The reason for midwives’ failure to consistently provide dietary assessment was that many assessments performed during ANC took a considerable amount of time. Prioritisation invariably leads to dietary assessment and nutrition education conducted last when time allows. However, nutrition education and counselling were prioritised in areas with nutrition issues. The importance of infant feeding and promotion of exclusive breastfeeding was provided by only a few (17.4%) midwives. This practice is of significant concern given the low breastfeeding rates of around 20% in Botswana, as per the 2020 Global Nutrition Report (2020). A study to determine health professionals’ effectiveness in nutrition education during pregnancy revealed that the timely provision of nutrition education to pregnant women played a vital role in a successful pregnancy (Teweldemedhin et al. 2021). Pregnant women mainly relied on midwives to guide and encourage healthy eating patterns; therefore, timely provision of good quality maternal nutrition remains a priority in Botswana.

The study had several strengths and limitations. The study setting comprised three different districts, giving a diverse and broad picture of midwives’ knowledge and practices. The study method involved both direct observations and administered interviewer questionnaires ensuring triangulation. Direct observations provided the opportunity to observe the actual practice conducted, which qualified the information given during the interview. However, the possibility of the hawthorn effect because of the availability of the researcher could not be ruled out. The unforeseen repeated lockdowns because of the coronavirus disease 2019 (COVID-19) pandemic brought a major limitation, prolonging the data-collection period. The entire data-collection process for the study took approximately 2 months between March and June 2020. The training for COVID-19 preparedness consequently reduced the population target for the study, as only a few midwives were available during the data-collection period.

### Recommendations

Further research on the midwifery curriculum is recommended focusing on the assessment of competent nutrition outcomes and level of priority allocated to maternal nutrition training in Botswana. There is a need to periodically provide on-the-job and refresher training on maternal nutrition to augment the insufficient training reported by the midwives. Developing and availing maternal nutrition guidelines and reference materials are also recommended as it will contribute to improved knowledge for the midwives. The guidelines will be used as a reference point and will fill the gap by increasing the knowledge needed to implement maternal nutrition services during ANC effectively.

## Conclusion

The study’s findings showed that maternal nutrition knowledge was inadequate to effectively provide maternal health services during ANC, as demonstrated by only 49% of midwives being knowledgeable on the subject matter despite the majority being more experienced. The observation that age was significantly associated with more knowledge highlights the importance of continuous professional education to ensure up-to-date information. However, their maternal nutrition practices were fairly performed, with an average overall score of 60% obtained in assessments during direct observation and practice interviews. Providing refresher training on maternal nutrition will likely improve the knowledge of nutrition of midwives and the nutrition services provided during ANC.
